# PTEN activation contributes to neuronal and synaptic engulfment by microglia in tauopathy

**DOI:** 10.1007/s00401-020-02151-9

**Published:** 2020-03-31

**Authors:** Joseph Benetatos, Rachel E. Bennett, Harrison T. Evans, Sevannah A. Ellis, Bradley T. Hyman, Liviu-Gabriel Bodea, Jürgen Götz

**Affiliations:** 1 Clem Jones Centre for Ageing Dementia Research, Queensland Brain Institute, The University of Queensland, Brisbane, QLD Australia; 2 Department of Neurology, MassGeneral Institute for Neurodegenerative Disease, Massachusetts General Hospital, Harvard Medical School, Charlestown, MA USA

**Keywords:** Microglia, Neurodegeneration, Phagocytosis, PTEN, Synapse, Tau

## Abstract

Phosphatase and tensin homolog (PTEN) regulates synaptic density in development; however, whether PTEN also regulates synapse loss in a neurodegenerative disorder such as frontotemporal lobar degeneration with Tau deposition (FTLD-Tau) has not been explored. Here, we found that pathological Tau promotes early activation of PTEN, which precedes apoptotic caspase-3 cleavage in the rTg4510 mouse model of FTLD-Tau. We further demonstrate increased synaptic and neuronal exposure of the apoptotic signal phosphatidylserine that tags neuronal structures for microglial uptake, thereby linking PTEN activation to synaptic and neuronal structure elimination. By applying pharmacological inhibition of PTEN's protein phosphatase activity, we observed that microglial uptake can be decreased in Tau transgenic mice. Finally, we reveal a dichotomous relationship between PTEN activation and age in FTLD-Tau patients and healthy controls. Together, our findings suggest that in tauopathy, PTEN has a role in the synaptotoxicity of pathological Tau and promotes microglial removal of affected neuronal structures.

**Electronic supplementary material:**

The online version of this article (10.1007/s00401-020-02151-9) contains supplementary material, which is available to authorized users.

## Introduction

Synapse loss is the best cellular correlate of impaired cognitive function in primary tauopathies, such as frontotemporal lobar degeneration-Tau (FTLD-Tau), and Alzheimer’s disease (AD) as the major secondary tauopathy [43, 52]. Despite this, our understanding of the process by which synapse loss occurs is limited [46]. In tauopathies, the normal axonal enrichment of Tau becomes disrupted and pathologically phosphorylated Tau progressively accumulates in the somatodendritic compartment, including synapses [19, 31, 58].

Because there is evidence that synapse loss, including that in tauopathies, involves intracellular components of apoptosis [45], we investigated the role of the lipid and protein phosphatase PTEN in this process. PTEN has been linked to neuronal apoptosis [13] and more specifically to the homeostatic regulation of dendritic spine numbers, in that a loss of PTEN causes excessive overgrowth of spines [29], whereas its overexpression results in their dramatic loss [40, 67]. PTEN activity is regulated by phosphorylation at serine (S) and threonine (T) residues which are located in its carboxyterminal domain. The enzyme further presents with a PDZ-binding domain that facilitates the interaction with adaptor proteins, such as postsynaptic density protein 95 (PSD-95) [24, 28, 67].

In development, PTEN regulates neuronal density in an activity-dependent manner, and blocking PTEN in neurons with reduced activity prevents their apoptotic death [64]. Synaptic removal has further been demonstrated to involve microglial cells, both in development [42, 54] and in Tau and amyloid-β (Aβ) mouse models of AD [10, 18]. Synapse engulfment by microglia requires extracellular molecules that are linked to apoptosis, such as phosphatidylserine (PS) and the complement factor C1q [16, 18]. PTEN has been shown to impair synaptic function in the APP/PSEN1 mouse model [28], but it has not been directly linked to synaptic elimination mediated by microglia. Taken together, this led us to hypothesize that synaptic and neuronal removal in disease may be mediated by a mechanism involving PTEN.

Here, we show that PTEN activation occurs early in disease pathogenesis in the rTg4510 and K3 tauopathy mouse models, whereas with advanced pathology, PTEN activation drops, similarly to what we observed in human FTLD-Tau patient brains. During the period of high PTEN activation, synapses and neuronal structures of rTg4510 mice increasingly expose the apoptotic signal PS, accumulate C1q and are engulfed by microglia. Pharmacological inhibition of PTEN in rTg4510 mice significantly reduces synaptic phagocytosis by microglia, preventing the loss of synaptic PSD-95 as well as preserving neuronal density in the hippocampus without affecting the level or phosphorylation of Tau, thereby placing PTEN activation downstream of Tau. Together, our data present PTEN as a mediator of tauopathy by facilitating the elimination of synapses and neuronal structures by microglia.

## Materials and methods

### Mice

Animal experimentation was approved by the Animal Ethics Committee of the University of Queensland (UQ) (approval numbers QBI/348/17/NHMRC and QBI/554/17/NHMRC) and by the Centre for Comparative Medicine (CCM) at Massachusetts General Hospital (MGH), in accordance with the National Institutes of Health Guide for the Care and Use of Laboratory Animals. The animals were housed in specific pathogen-free cages and maintained on a 12 h light/dark cycle, with constant access to food and water. Transgenic rTg4510 mice express human four-repeat Tau with the P301L mutation linked to hereditary tauopathy [41]. Male mice were used unless otherwise stated in the methods section. Mouse tissue extracts were prepared at UQ, with the exception of tissue from 15 and 18 month-old wild-type and rTg4510 mice which were prepared at MGH. K3 mice express the K369I mutation of Tau under the control of the mThy1.2 promoter [21], and Cx3Cr1^eGFP/−^ mice express EGFP under the control of the microglia-specific Cx3Cr1 promoter [23]. For both K3 and Cx3Cr1^eGFP/−^ strains, mice from both genders were used. APP23 mice overexpress human mutant amyloid precursor protein harboring the Swedish mutation [55].

### Human samples

Autopsy tissue from FTLD-Tau and healthy-control cases from MGH were collected with informed consent of patients or their relatives and after approval of local institutional review boards has been obtained. Human patient demographic information for control and FTLD-Tau is provided in Table 1. All experimentation with human tissue was performed at MGH.

**Table 1:** Human cases used for PTEN analysis

#	Diagnosis	Chromosomal abnormality	Braak	Age	Gender	Post mortem interval
1	Control		II	90 +	F	24
2	Control		I	68	M	27
3	Control		I	98	M	24
4	Control			86	M	10
5	Control		0	90 +	m	21
6	Dementia lacking distinctive histology (DLDH)		I	78	F	6
7	Control		I	90 +	F	8
8	Frontotemporal lobar degeneration with Tau pathology (FTLD-tau)	P301L	IV	71	f	4
9	Frontotemporal lobar degeneration with Tau pathology (FTLD-tau)	P301L		56	M	
10	Corticobasal degeneration (CBD)	P301L	0	61	M	14
11	Pick's disease type A (PiD-A)	P301L		70	M	12
12	Pick's disease (PiD)	P301L		33	M	33
13	Frontotemporal lobar degeneration (FTLD-TAU)		0	70	M	2
14	Frontotemporal lobar degeneration with Tau pathology (FTLD-tau)			71	F	12
15	Frontotemporal lobar degeneration with Tau pathology (FTLD-tau)			70	F	12
16	Frontotemporal lobar degeneration with Tau pathology (FTLD-tau)			90 +	F	

### Primary neuronal cultures

Embryonic day (E)17 cortical and hippocampal neurons were obtained from rTg4510 and non-transgenic (WT) embryos and plated onto poly-d-lysine (PDL)-coated coverslips in a 24-well plate, at a density of 30,000 cells/well. For their culture, Neurobasal medium (Gibco) was supplemented with 5% fetal bovine serum (Hyclone), 2% B27 (Gibco), 2 mM Glutamax (Gibco) and 50 U/ml penicillin/streptomycin (Invitrogen). The medium was changed to serum-free Neurobasal medium 24 h post-seeding and half of the medium was further changed twice a week.

### Primary microglial/neuronal co-culture

Cortices were dissected from postnatal day 3 Cx3Cr1^eGFP/−^ pups. Cortices were plated into flasks containing DMEM-F12 (Gibco) supplemented with 50 U/ml penicillin/streptomycin (Invitrogen), 2 mM Glutamax (Gibco) and 0.45% glucose (Sigma). The medium was changed 24 h after plating and then every 5 days. Day in vitro (DIV) 21–28 microglia were isolated by placing the flasks for 3 h in an orbital shaker in a 37 °C incubator at 450 rpm. Supernatants were collected and filtered through a 70 μm cell strainer, followed by centrifugation at 200 rpm to obtain the microglial pellet. For replating on primary neurons, the microglial pellet was resuspended in medium supplemented with Neurobasal, adding 30,000 microglia to 30,000 neurons maintained in a 24-well plate.

For the microglial phagocytosis assay, microglia were resuspended in DMEM-F12 and plated into a PDL-coated black, clear bottom 96-well plate at a density of 10,000 microglia/well.

### Synaptosomal and PSD fraction preparation

Subcellular fractionation was performed with minor modifications as described previously [35]. rTg4510 mice were deeply anesthetized with sodium pentobarbital and brains were extracted following perfusion with PBS. Brain tissue was first homogenized on ice in sucrose buffer (0.32 M sucrose, 10 mM HEPES, pH 7.4), and the total brain homogenate was centrifuged at 1000×*g* for 10 min at 4 °C, yielding the supernatant fraction or total protein (TP) and the nuclear enriched pellet (P1). The supernatant was centrifuged at 14,000*g* for 20 min at 4 °C to obtain the crude synaptosomal fraction (P2) and the cytosolic protein enriched supernatant (Cyto). The P2 pellet was washed twice with wash buffer (4 mM HEPES, 1 mM EDTA, pH 7.4) by resuspension and centrifugation at 12,000×*g* for 20 min at 4 °C and then resuspended in buffer A (20 mM HEPES, 100 mM NaCl, 0.5% Triton X‐100, pH 7.2). After rotation at 4 °C for 1 h, the suspension was centrifuged at 12,000×*g* for 20 min at 4 °C to yield the non‐PSD fraction containing extra-synaptic proteins. The resultant pellet was washed twice in wash buffer and then resuspended in buffer B (20 mM HEPES, 0.15 mM NaCl, 1% Triton X‐100, 1% SDS, 1 mM dithiothreitol, 1% deoxycholate, pH 7.5) for 1 h at 4 °C, followed by centrifugation at 10,000×*g* for 20 min at 4 °C to obtain the PSD fraction containing synaptic proteins. All buffers were freshly supplemented with protease and phosphatase inhibitor cocktail (Cell Signaling Technology) prior to use, and fractions were stored as aliquots at − 80 °C.

### In vitro engulfment assay

To obtain pHrodo-conjugated synaptosomes, synaptosomes purified from rTg4510 and WT brains were incubated at room temperature (RT) in isotonic buffer containing pHrodo-AM™ Red succinimidyl ester (Invitrogen) under gentle agitation. After 30 min incubation, unbound pHrodo dye was washed out by multiple rounds of centrifugation, and the pHrodo-conjugated synaptosomes were resuspended in isotonic buffer containing 10% DMSO and stored at − 30 °C until use. On the day of the assay, synaptosomes were thawed and diluted in live cell imaging solution and added in triplicate to a 96-well plate, with wells either containing DIV 21–28 primary microglia obtained from Cx3Cr1^eGFP/−^ mice, or no microglia. The 96-well plate was placed immediately into a fluorescence plate reader and the intensity from each well was obtained. This was repeated after 15 min and then again every 30 min for a total duration of 4.25 h. The plotted intensity was obtained by subtracting of wells without microglia from that of wells containing microglia.

### Western blot analysis of mouse tissue

Total brain and synaptosomes were solubilized in RIPA buffer supplemented with protease and phosphatase inhibitors followed by sonication, using a probe sonicator set of 20 s at a 20% power. Samples were centrifuged at 12 k rpm for 10 min at 4 °C. Protein (10–20 µg) from each sample was loaded onto a 4–15% Bis-Tris gels, transferred to a PVDF membrane and blocked for 1 h with Tris-buffered saline with Tween-20 (TBS-T) blocking buffer, and then incubated in primary antibodies at 1:1000 in TBS-T blocking buffer overnight. Then, membranes were washed 3 × 5 min in TBS-T. Secondary antibodies were added at 1:10,000 for 1 h in 50% TBS-T blocking buffer. A Li-Cor detection system was used for imaging. For reprobing, blots were stripped using Reblot plus strong antibody stripping solution (Merck) for 20 min at room temperature.

### Western blot analysis of human tissue

Each sample of 100 mg of human frontal cortex tissue (Brodmann Area 10) was dounce homogenized in RIPA containing protease (cOmplete Mini Protease Inhibitor Cocktail, Roche) and phosphatase (PhosSTOP, Roche). Homogenates were spun at 10,000 × g for 10 min at 4 °C to remove insoluble debris, and the resulting supernatant was reserved for Western blotting. 4–12% pre-cast SDS polyacrylamide gels (Invitrogen) were loaded with 10 µg protein per well and run at 120 V for 90 min in MES buffer (Invitrogen). Transfer to 0.2 µm pore size nitrocellulose membrane took place at 90 V for 90 min. The resulting membranes were rinsed briefly in TBS (blots for pTau were boiled for 3 min in PBS) and then blocked for 1 h in Li-Cor blocking buffer. Primary antibodies were applied overnight in blocking buffer. After rinsing in TBS, secondary infrared antibodies were applied the next day in blocking buffer (Li-Cor, 1:5000) and then imaged using an Odyssey CLX infrared imager. Blots were then stripped using NewBlot Nitrocellulose stripping buffer (Li-Cor) for 10 min at room temperature.

### Validation of pPTEN signal on western blot using phosphatase treatment

Total brains from WT mice were solubilized in RIPA buffer supplemented with protease inhibitors followed by sonication, with a probe sonicator set of 20 s at a 20% power. Samples were centrifuged at 12 krpm for 10 min at 4 °C. Samples were then treated with lambda protein phosphatase according to the manufacturers' protocol (NEB). Protein samples were incubated with NEBuffer for protein metal-dependent phosphatases and MnCl_2_ and increasing units of lambda protein phosphatase at 37 °C for 30 min. Western blot and analysis were then performed as described above.

### Immunofluorescence labeling

For histology, mice were deeply anesthetized with sodium pentobarbital and brains were extracted following perfusion with PBS. Tissue was placed in 4% paraformaldehyde (PFA) for 24 h, and then transferred to a 30% sucrose solution in PBS. Once the brains were equilibrated, they were frozen on dry ice and sectioned on a Leica freezing sledge microtome at a thickness of 40 μm. Sections were stored at − 20 °C in cryoprotectant solution. Sections were blocked for 1 h at RT in 10% BSA in PBS with 0.2% Triton X-100. Primary antibodies were diluted in blocking buffer at concentrations ranging from 1:100 to 1:1000 and sections were incubated at 4 °C in primary solution for 24–72 h. After incubating in primary solution, sections were washed 3 × for 5 min at RT in PBS. This was followed by a 1 h incubation with secondary antibodies conjugated to the appropriate fluorophores in PBS with 0.2% Triton-X. Sections were then washed in PBS with 4′,6-diamidino-2-phenylindole (DAPI) (1:10,000) for 5 min at RT, followed by 2 washes in PBS for 5 min each at RT. They were then mounted on slides, and cover slipped using Vectashield hard-set mounting medium.

### Tissue imaging and analysis

The tissue was imaged on a spinning-disk confocal system (Marianas; 3I, Inc.) consisting of an Axio Observer Z1 (Carl Zeiss) equipped with a CSU-W1 spinning-disk head (Yokogawa Corporation of America), ORCA-Flash4.0 v2 sCMOS camera (Hamamatsu Photonics), 63 × 1.4 NA Plan-Apochromat oil immersion objective. Image acquisition was performed using SlideBook 6.0 (3I, Inc.). Images consisting of 78 z-stacks at 0.13 μm steps were obtained for images analyzed.

For the in vivo volume of CD68 analysis, a surface rendering was created for the channel being analyzed and the volume was quantified.

For the in vivo analysis of colocalization, image were deconvoluted in Huygens Pro. Using the Imaris software, a spots function was created for each channel, and the colocalized spots were counted using a distance cut-off of 100 nm between the edges of the spots.

For the in vivo percent area analysis, microglia numbers, and mean gray value images were analyzed with ImageJ software.

For in vivo engulfment analyses of PSD-95 and MAP2, a surface rendering was first created for CD68 and then used to mask the channel being measured for engulfment, all pixels outside the surface being set to 0. A surface was then created for the masked channel and the volume of the surface was quantified.

For the in vivo analysis of the PTEN mean gray value within pTau positive and pTau negative areas, a maximum intensity z-stack of the image was created. The corresponding pTau channel was thresholded and a mask was created and added to the region of interest (ROI) manager. The pTau mask was then added to the PTEN channel, the area outside the mask was cleared for assessment of PTEN mean gray value within Tau positive areas, and the area inside the mask was cleared for assessment of PTEN mean gray value in Tau negative areas.

### Flow cytometry

The synaptosome-containing pellet (above) was resuspended in binding buffer (10 mM HEPES pH 7.4), 150 mM NaCl, 5 mM KCl, 5 mM MgCl_2_, 1.8 mM CaCl_2_), after which 100 μl aliquots were incubated for 30 min at 20–25 °C with dye solution and then diluted in PBS (final volume, 1000 μl) for immediate flow cytometry analysis. Final dye concentration was 1 μM for Calcein AM Blue (Invitrogen) and 5 μl/ml for p-SIVA (Novus Bio.). The synaptosomes were analyzed on a BD FACSAria Cell Sorter.

### Live imaging of membrane asymmetry

Cortical neurons were plated on live cell imaging dishes with four chambers. On DIV20, Neurobasal medium was removed and replaced with 1 ml pre-warmed Live Cell imaging solution (Gibco) with 1 µl F2N12S membrane asymmetry dye (ThermoFisher). Neurons were incubated at 37 °C for 5 min before imaging using an LSM 710 Zeiss confocal microscope. The dye was excited using a 405 nm laser line, with emission simultaneously being captured at 535 and 585 nm, using dual camera acquisition. 5 separate XY coordinates were programmed and imaged sequentially 5 times. Images were analyzed using the Ratio Plus plugin in ImageJ. Each data point is the average of 5 images taken at each XY coordinate.

### Pharmacological inhibition of PTEN

Mice from both genders were randomized and separated into groups of five. The mice received intraperitoneal injections of the PTEN inhibitor dipotassium bisperoxovanadium(pic) dihydrate (bpv(pic)) (Merck) at 1 mg/kg, or 0.9% saline twice weekly for 5 months.

## Reagents

A list of critical reagents and resources can be found in Supplementary Table 1.

### Statistical analysis

All statistical analyses were performed using GraphPad Prism 7, with statistical tests used as per figure legend. Statistical significance was defined as: n.s.—not significant, *p < 0.05, **p < 0.01, ***p < 0.001, ****p < 0.0001. Data throughout the paper are displayed as mean ± SEM. Experimenters were blinded whenever possible to experimental condition during data acquisition or quantification.

## Results

### Increased caspase-3 cleavage and exposure of ‘eat-me’ signals in tauopathy

**Fig. 1:**
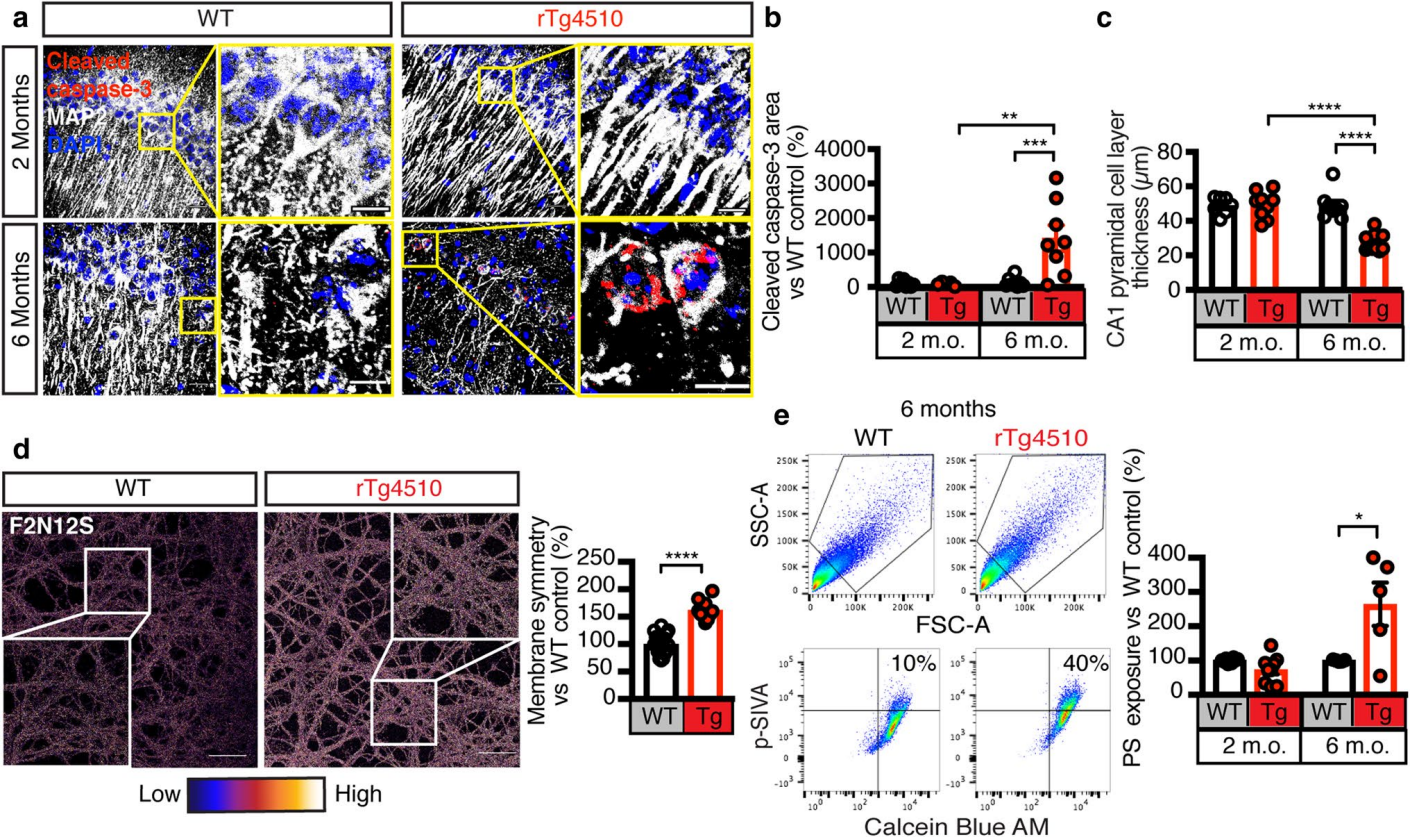
Increased apoptotic hallmarks cleaved caspase-3 and phosphatidylserine exposure in rTg4510 mice. **a–c** Maximum intensity z-projection images of the hippocampus of 2 and 6 month-old rTg4510 and WT mice probed with the apoptosis marker Cleaved caspase-3 (red), the neuronal marker MAP2 (white) and the nuclear marker DAPI (blue). Quantification of the images reveals a massive increase in neuronal apoptosis and cell loss in rTg4510 mice at 6 months. Scale bars: 20 μm. **d** Representative images and quantification of WT and rTg4510 primary neurons labeled with the membrane asymmetry probe F2N12S. A higher ratio (pseudo-colored as low-blue to high-white) indicates more membrane symmetry and correlates with the exposure of negatively charged phospholipids including PS on the surface. This provides evidence that there is more PS exposure in rTg4510 neurons. Inset is zoomed-in image of white framed region. Scale bars: 10 μm. **e** Representative plots and quantification of the flow cytometry analysis of apoptotic PS exposure (p-SIVA^+^) on viable (Calcein Blue AM^+^) synaptosomes obtained from 2 and 6 month-old WT and rTg4510 mice. This analysis indicates that when comparing equal fractions of viable synaptosomes, there is a greater proportion of apoptotic synaptosomes in rTg4510 than WT mice. Unpaired *t* test within the same age group. Data are presented as mean ± SEM, *p < 0.05, **p < 0.01, ***p < 0.001, ****p < 0.0001; **b**, **c** two-way ANOVA with Tukey’s multiple comparison test, **e**, **f** unpaired *t* test

PTEN functions as a tumor suppressor to promote apoptosis by antagonizing the pro-survival pathway [30]. We therefore sought to determine whether apoptosis was occurring in the rTg4510 mouse strain that expresses human Tau with the P301L FTLD-Tau mutation and presents with age-dependent progressive Tau pathology [41]. We assessed rTg4510 and wild-type (WT) littermate control mice at 2 and 6 months for the presence of cleaved caspase-3, an end stage marker of apoptosis [12]. We found increased neural apoptosis at 6 months of age, as evidenced by cleaved caspase-3-positivity in CA1 pyramidal cell layer neurons that were identified using the neuronal marker microtubule-associated protein 2 (MAP2) (Fig. 1a–c). Accelerated apoptosis was also apparent in primary cortical neurons obtained from rTg4510 mice (Fig. S1a).

During apoptosis, plasma membranes lose their molecular asymmetry through a mechanism by which PS, a negatively charged amine-containing phospholipid, becomes externalized to the outer leaflet of the cell membrane [34]. We therefore sought to determine whether PS is exposed outside of the cell membrane in tauopathy by probing rTg4510 and WT primary neurons with the membrane asymmetry probe F2N12S, which responds to changes in the charge of the membrane following PS exposure [9, 11, 20, 25, 49]. Live imaging of rTg4510 compared to WT primary neurons labeled with F2N12S revealed a pronounced gain in membrane symmetry, indicating increased exposure of PS in the presence of pathological Tau (Fig. 1d).

Having determined that primary neurons obtained from rTg4510 mice expose PS, we next sought to determine whether this exposure is also found at the synapse. We therefore isolated synaptosomes from rTg4510 and WT mice and incubated them with both the membrane-impermeable marker p-SIVA, a polarity-sensitive annexin probe that labels exposed PS [26], and the membrane-permeable dye Calcein Blue AM, which becomes fluorescently active inside sealed and intact membranes [8]. Our results revealed no detectable difference between the two genotypes at 2 months of age; however, at 6 months of age, rTg4510 mice displayed an increase in synaptosomes with a high fluorescence signal for both p-SIVA and Calcein AM Blue (Fig. 1e).

**Fig. 2:**
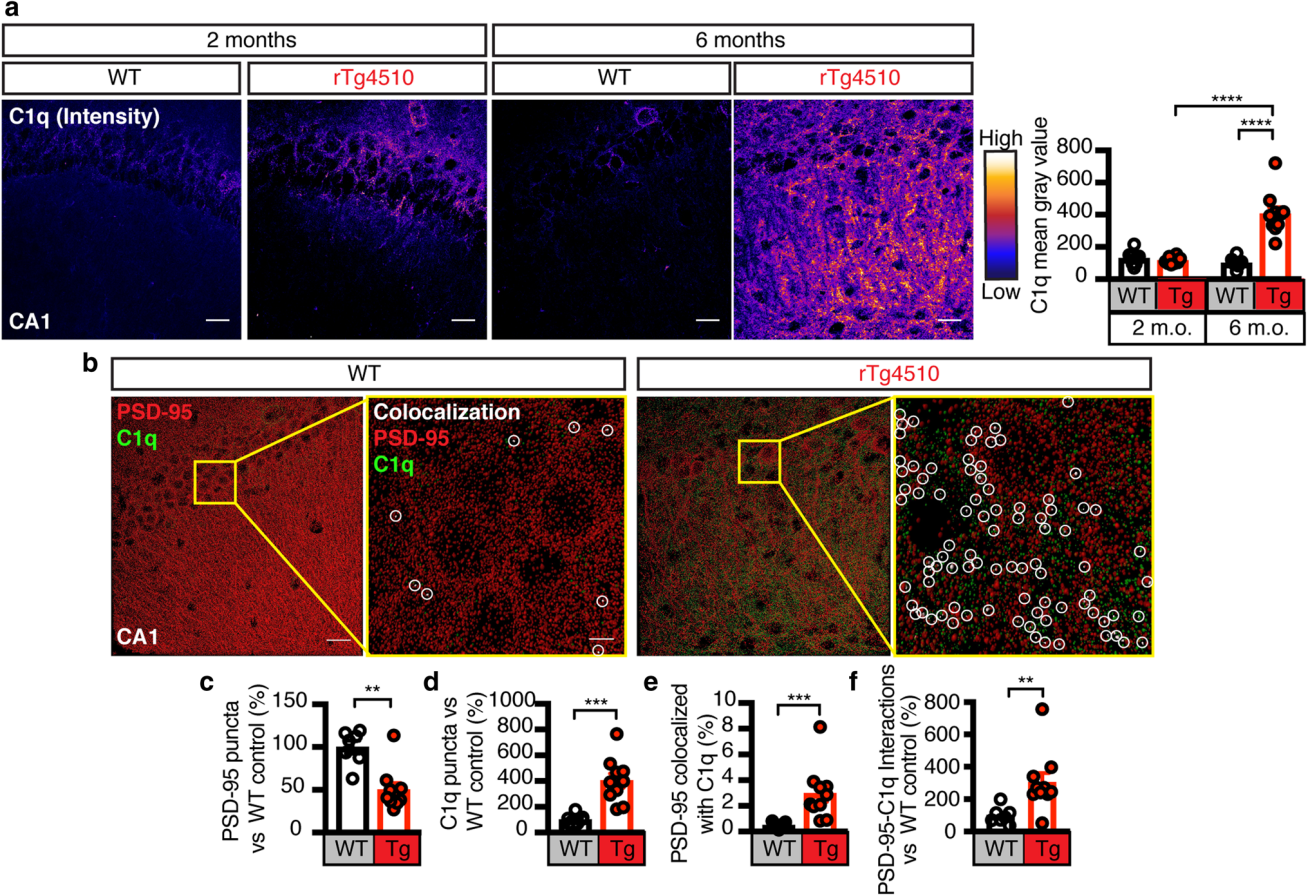
Increase of complement factor C1q and its association with PSD-95 in rTg4510 mice. **a** Representative fluorescence images and analysis for C1q (pseudo-colored from low-blue to high-white) from the CA1 region of 2 and 6-month-old rTg4510 and WT mice. The analysis reveals an increase in C1q intensity between 2 and 6 months in rTg4510 mice and an increase between rTg4510 and WT mice at 6 months. **b** 3D spots rendering of PSD-95 (red), and C1q (green) from (C) following deconvolution. Zoomed-in images with yellow border display colocalization of PSD-95 and C1q in white (circled). Scale bar: 20 μm; zoomed-in image: 5 μm. **c** Quantification of the number of PSD-95 puncta in the WT and rTg4510 hippocampus, showing a significant loss in PSD-95 occurring in rTg4510 mice at 6 months of age. **d** Quantification of C1q puncta between WT and rTg4510 mice, indicating a large increase in the total number of C1q puncta in rTg4510 mice. **e** Quantification of the fraction of total PSD-95 tagged with C1q, indicating that despite a loss of PSD-95, there is a greater fraction of PSD-95 that is associated with C1q. **f** Analysis of the number of colocalized PSD-95/C1q puncta demonstrating that in rTg4510 mice more PSD-95 is tagged with the “eat-me” signal C1q. Data presented as mean ± SEM, *p < 0.05, **p < 0.01, ***p < 0.001, ****p < 0.0001; **a** two-way ANOVA with Tukey’s multiple comparison test, **c**, **d**, **e**, **f** unpaired *t* test

PS exposure can be detected in the brain via the ‘eat-me’ signal C1q that binds to PS [16, 37, 56, 63]. We found that the C1q intensity in the hippocampus of rTg4510 mice was significantly increased between 2 and 6 months of age, an effect that was not observed in WT mice (Fig. 2a). Automated counting and analysis of C1q puncta in deconvoluted confocal images confirmed an increase in rTg4510 mice (Fig. 2b, d). We also observed an increase in the fraction of PSD-95 co-localized with C1q in these mice (Fig. 2e). Despite the decrease in numbers of PSD-95 puncta that reflects the synapse loss seen in this mouse strain (Fig. 2c), the total number of PSD-95/C1q interactions was increased (Fig. 2f). Together, we revealed that Tau promotes the exposure of PS, which facilitates the removal of neuronal structures and synapses by microglia.

### Increased microglial engulfment of synapses and neuronal structures in rTg4510 mice

**Fig. 3:**
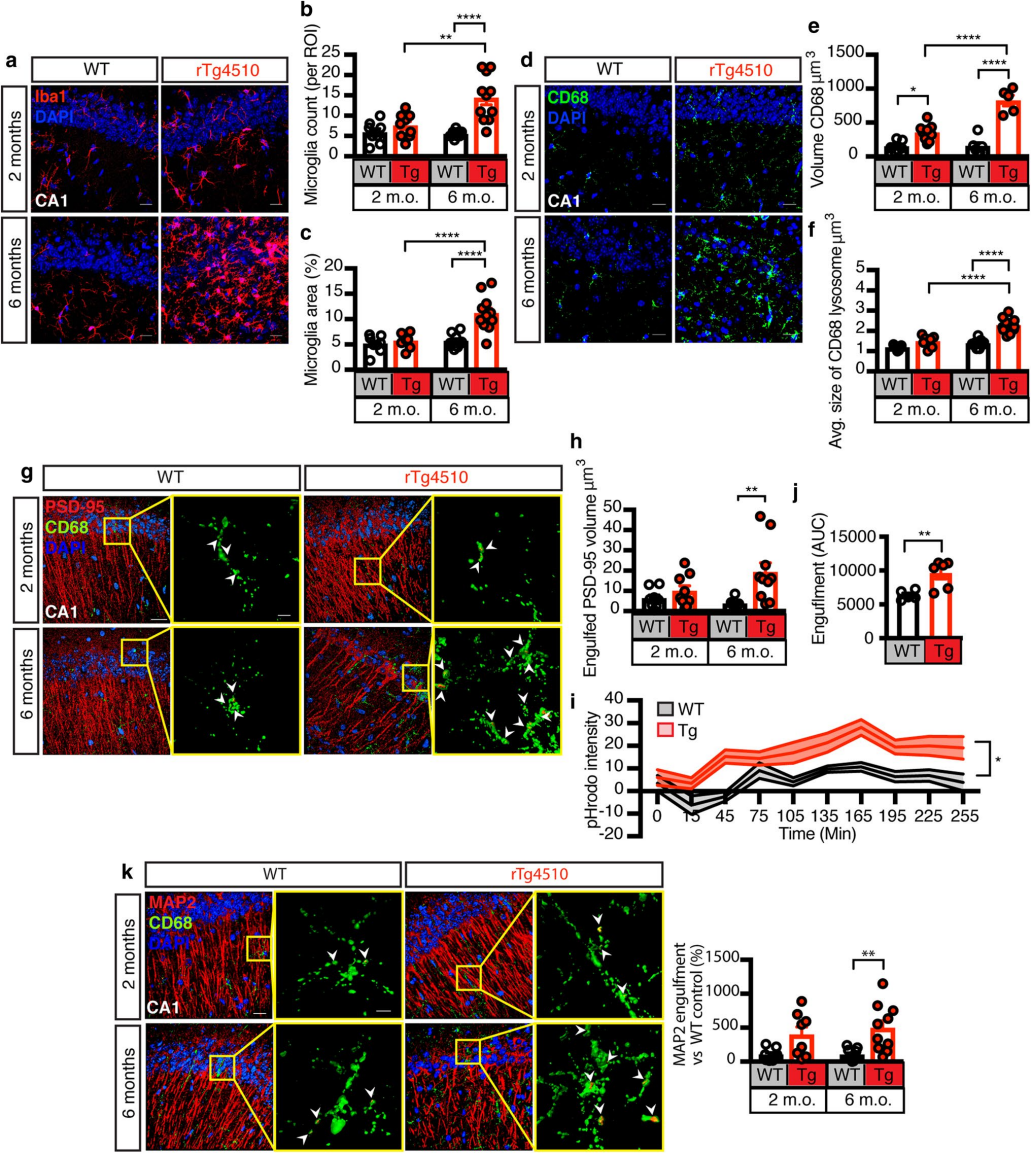
Microglia increasingly engulf neurons and synapses in rTg4150 mice. **a** Representative fluorescence maximum intensity z-projections for the microglial marker Iba1 (red) and the nuclear marker DAPI (blue) in the CA1 region of 2 and 6 month-old WT and rTg4510 mice. Scale bar: 20 μm. **b** Quantification of microglia per ROI from **a**, counting DAPI- and Iba1-positive cells. This analysis provides evidence of microgliosis in rTg4510 mice. **c** Quantification of microglia area per ROI from **a**, analyzed by thresholding the Iba1 signal. Together with **b** this analysis provides evidence of microgliosis in rTg4510 mice. **d** Representative fluorescence maximum intensity z-projections of the microglial lysosomal marker CD68 (green) and DAPI (blue) in the CA1 region of 2 and 6 month-old WT and rTg4510 mice. Scale bar: 20 μm. **e** Quantification of the volume of CD68 from (D), measured by surface rendering in Imaris. This analysis demonstrates that microglial lysosome production, a proxy for phagocytic activity, is increased in rTg4510 mice. **f** Quantification of the average volume of individual CD68 lysosomes from (D), measured by surface rendering in Imaris. **g** 3D rendering of the postsynaptic marker PSD-95 (red), CD68 (green) and DAPI (blue), with arrowheads indicating engulfment. **h** Quantification of the volume of PSD-95 engulfed by microglia in the hippocampus of 2 and 6 month-old rTg4510 and WT mice. This analysis provides evidence of microglia engulfment of synaptic structures in rTg4510 mice. Scale bar in composite image: 20 μm; scale bar in zoomed-in images: 5 μm. **i** Time course quantification of synaptosomal engulfment by microglia using the acidification sensor pHrodo-AM red. Data points at each time segment represent individual animals analyzed in triplicates. This analysis demonstrates that synaptosomes from rTg4510 mice are primed for engulfment by microglia. **j** Quantification of the area under the curve from **i**, representative of total engulfment. **k** 3D rendering of the neuronal marker MAP2 (red), CD68 (green) and DAPI (blue), with arrowheads indicating engulfment. Quantification of MAP2 engulfment by microglia in the hippocampus of 2 and 6 month-old rTg4510 and WT mice. This analysis provides evidence of microglia engulfment of neuronal structures in rTg4510 mice. Scale bar in composite image: 20 μm; scale bar in zoomed-in images: 5 μm. Data presented as mean ± SEM, *p < 0.05, **p < 0.01, ****p < 0.0001; **b**, **c**, **e**, **f**, **h**, **i** and **k** two-way ANOVA with Tukey’s multiple comparison test, **j** unpaired *t* test

To understand how microglia respond to the pathological Tau built-up in the rTg4510 hippocampus, we counted the number of microglia and the percentage area covered by them in comparison to WT mice. At 2 months of age, these parameters did not differ between the two genotypes; at 6 months, however, rTg4510 mice had significantly more microglia per region of interest (ROI), compared to 2 month-old rTg4510 and 6 month-old WT mice (Fig. 3a–c). We next investigated microglial phagocytosis by assessing the microglial lysosomal marker Cluster of Differentiation 68 (CD68) and found an increased volume in the hippocampus of both 2 and 6 month-old rTg4510 mice compared to WT controls (Fig. 3d, e). The average size of these lysosomes was increased in the rTg4510 hippocampus compared to WT mice at 6 months of age and they were significantly larger than those of 2 month-old rTg4510 mice (Fig. 3f).

We next used spinning-disk confocal microscopy followed by surface rendering to determine whether PSD-95 was internalized into CD68-labeled lysosomes. We found that the volume of engulfed PSD-95 structures was increased in the hippocampus of 6 month-old rTg4510 mice compared to WT controls (Fig. 3g). To assess to which extent synaptosomes were internalized by microglia, we incubated rTg4510 and WT synaptosomes with pHrodo-AM, a dye that fluoresces in an acidic environment such as the lysosome, and added them to primary EGFP-labeled microglia isolated from Cx3Cr1^eGFP/−^ mice. We found that the pHrodo intensity increased at a faster rate in the rTg4510 synaptosomes than in the WT samples, and remained at an elevated level throughout the ~ 4 h of the experiment (Fig. 3i). Total engulfment was also assessed by measuring the area under the curve, which revealed that microglia engulfed more synaptosomes from rTg4510 than WT mice (Fig. 3j). We reasoned that the engulfment of neuronal structures by microglia may not be limited to the postsynaptic density (PSD). To confirm this, we repeated the in vivo engulfment measurements using MAP2 as a proxy for neuronal structures that may be taken up in addition or independently of PSD-95. We found that microglia in the rTg4510 had increased MAP2 structures located within CD68-labeled lysosomes compared to WT controls (Fig. 3k).

### PTEN activation precedes caspase-3 cleavage, with levels correlating with those of pTau

**Fig. 4:**
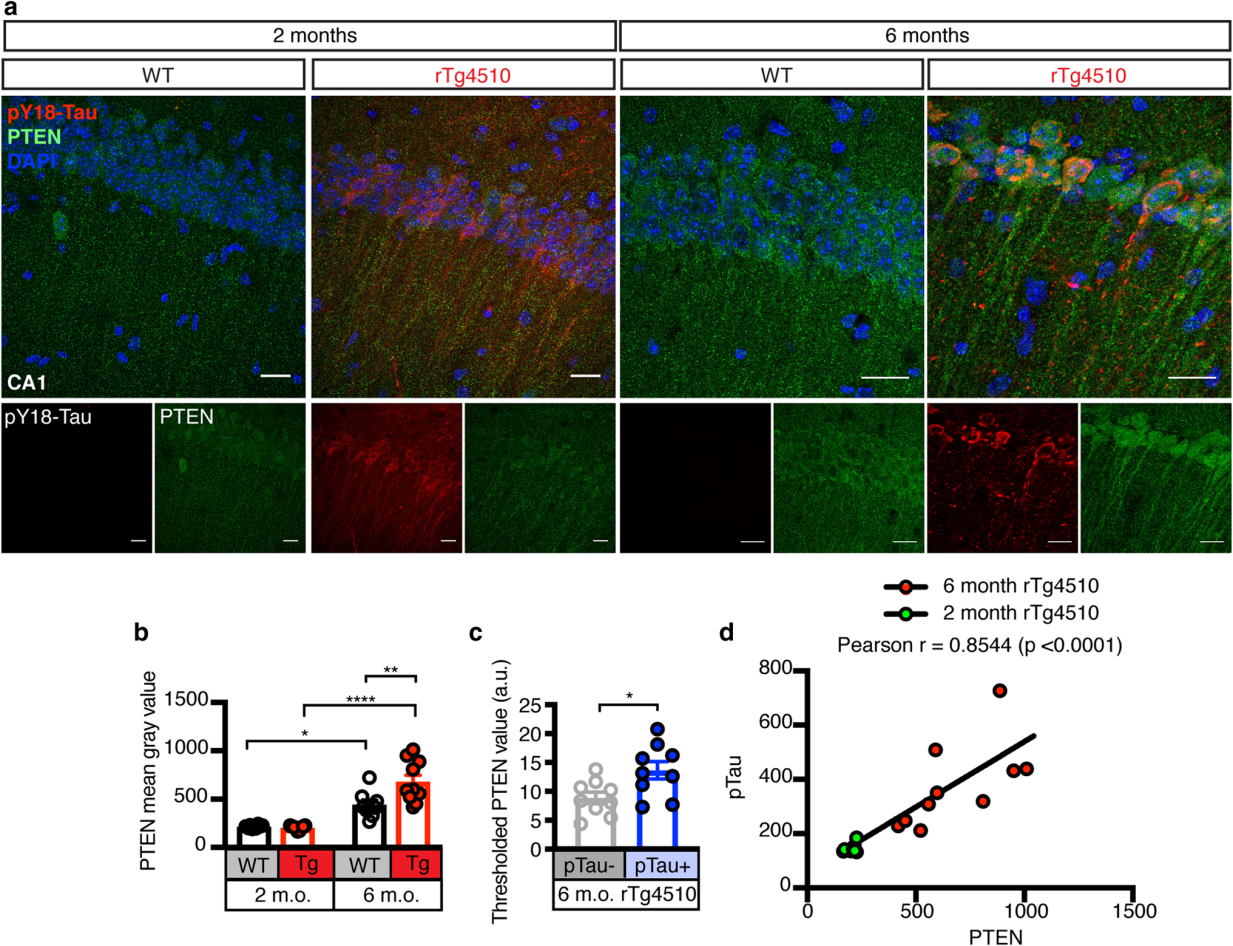
PTEN levels increase and correlate with pTau in the hippocampus of rTg4510 mice. **a** Representative fluorescence images for Tau phosphorylated at Tyr18 (pY18 Tau, red), PTEN (green), and DAPI (blue) obtained from 10 μm thick maximum intensity z-projections of the hippocampus (CA1 region) of 2 and 6 month-old Tau P301L transgenic rTg4510 and WT mice. Scale bar: 20 μm. **b** Quantification of the PTEN mean gray value in the hippocampus of 2 and 6 month-old rTg4510 and WT mice, demonstrating that PTEN levels increase with age in rTg4510 mice. **c** Quantification of the PTEN mean gray value in pTau- and pTau + areas of 6 month-old rTg4510 mice, indicating enrichment of PTEN in neurons that are accumulating pTau. **d** Pearson correlation between the PTEN and pY18 Tau mean gray values revealing age-dependency. Data presented as mean ± SEM, *p < 0.05, **p < 0.01, ****p < 0.0001; **b** two-way ANOVA with Tukey’s multiple comparison test, **c** unpaired *t* test, **d** Pearson correlation

We next assessed the pathological phosphorylation of Tau (pTau) at the early epitope Tyrosine 18 (pY18) [2, 3, 36] in relation to PTEN expression and distribution in rTg4510 mice using immunofluorescence (Fig. 4a). This revealed increased PTEN levels in the hippocampus at 6, but not 2 months of age compared to WT littermate control mice (Fig. 4a, b). Interestingly, PTEN expression was increased with age in both genotypes (Fig. 4a, b). In relation to pY18 Tau, we revealed in 6 month-old rTg4510 mice that PTEN expression was higher in Tau-positive neurons compared to those which stained negative for pTau (Fig. 4c). To determine whether levels of pY18 Tau correlated with those of PTEN, we measured pY18 Tau intensities in the hippocampus of rTg4510 mice at 2 and 6 months of age and plotted them against PTEN, which revealed a positive correlation (Fig. 4d). This was also observed in the cortex, another region with Tau pathology in this mouse strain (Fig. S2a–c).

**Fig. 5:**
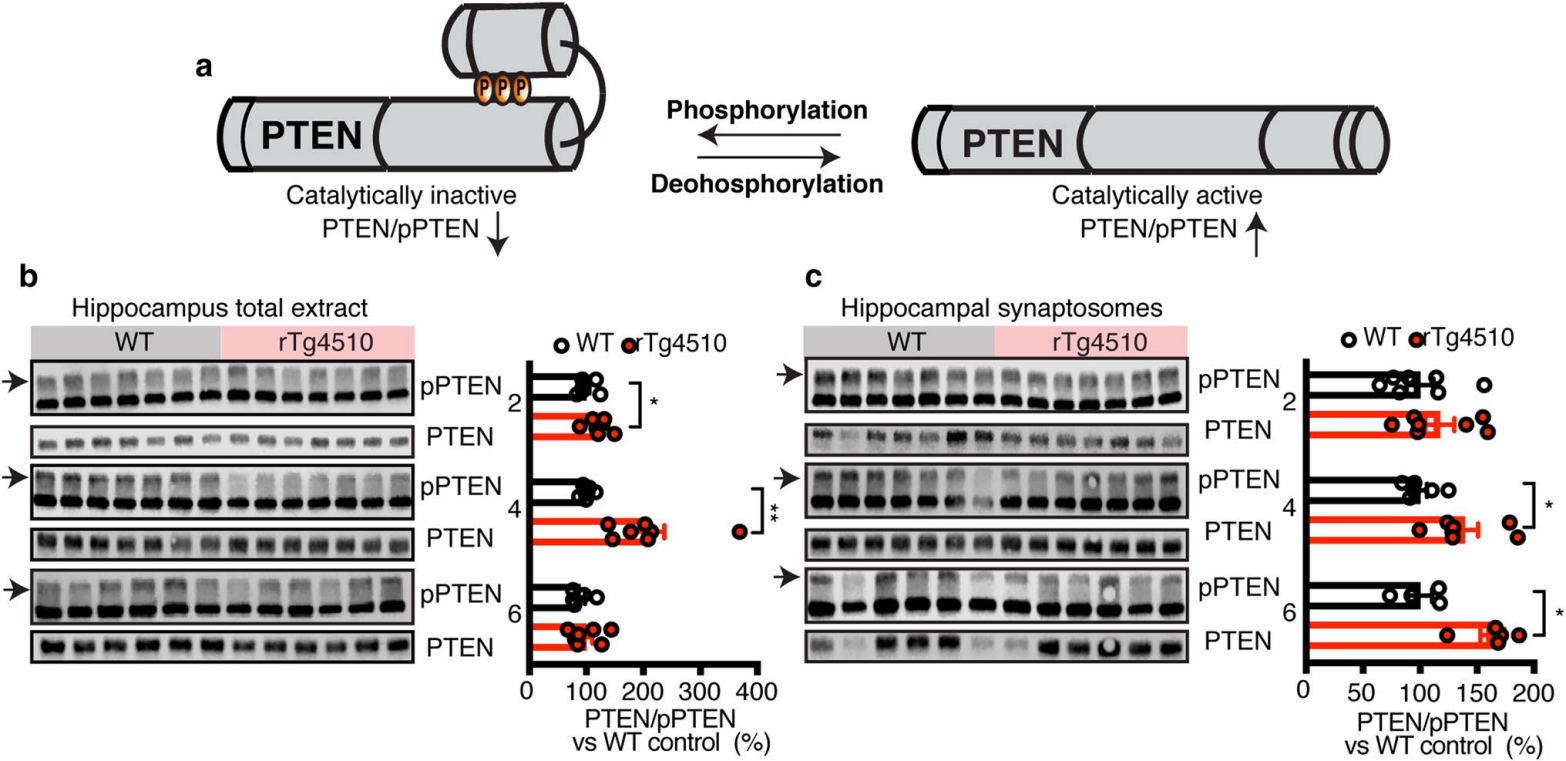
Increased PTEN activation in rTg4510 mice, preceding caspase-3 cleavage. **a** Schematic depicting negative regulation of PTEN activity by phosphorylation. **b** Representative western blots and quantification of pPTEN (inactive) and total PTEN using extracts from the hippocampus of 2, 4, and 6 month-old WT and rTg4510 mice reveal an increase in total PTEN levels relative to the inactive form at 2 and 4 months. **c** Representative western blots and quantification of pPTEN (inactive) and total PTEN of synaptosomal lysates from the hippocampus of 2, 4, and 6 month-old WT and rTg4510 mice, revealing an increase in total PTEN relative to the inactive form at 4 and 6 months. Data presented as mean ± SEM, *p < 0.05, **p < 0.01, **b**, **c** unpaired *t* test performed between WT and rTg4510 at each age group independently

Of the four critical residues that negatively regulate PTEN activity [4], only S380, T382 and T383 (together referred to here as pPTEN) are detectable by antibody-based methods. We assessed the ratio of total PTEN to pPTEN (i.e. inactive PTEN) (Fig. 5a) as a measure of activity in rTg4510 brains, with an increase in this ratio corresponding to an increase in activity. To validate the specificity of the pPTEN antibody in mouse samples, we incubated lysates with increasing concentrations of phosphatase, which identified the upper of the two bands (which was hence used for quantification) as being specific for pPTEN (Fig. S3a).

We analyzed total protein lysates from the hippocampus of rTg4510 and WT mice at three time points (2, 4, and 6 months), and observed an increased ratio of activated PTEN in rTg4510 compared to WT mice at 2 and 4 months of age (Fig. 5b). Similarly, total protein lysates from the cortex also revealed an age-dependent increase in PTEN activation (Figure S3b).

Under pathological conditions, Tau re-localizes to the somatodendritic compartment and also accumulates in the PSD where it exerts synaptotoxicity [10, 22]. We confirmed the presence of pathological Tau in the PSD at the earliest time-point using purified PSD fractions from 2 month-old rTg4510 and WT mice, and analyzed total and pY18 Tau levels by immunoblotting. We found abundant levels of pY18 Tau in both the hippocampal and cortical PSD fractions (Fig. S3c). We next analyzed PTEN activation in synaptosomal fractions (which contain both the pre- and post-synapse) from the hippocampus of 2, 4, and 6 month-old WT and rTg4510 mice. Immunoblotting of total and phosphorylated PTEN revealed an increase in PTEN activity in rTg4510 compared to WT in 4 and 6 month-old mice (Fig. 5c), a pattern also found in cortical synaptosomal fractions (Figure S3d).

To determine whether PTEN is also activated in a tauopathy model with a much less pronounced pathology, we examined cortical brain lysates from the K3 mouse strain, which expresses human Tau together with the FTLD-Tau mutation K369I [21]. These mice present with widespread Tau pathology in the cortex [38]. We found increased PTEN activation in total cortical lysates from 12 month-old K3 mice compared to WT animals (Figure S3e). We next sought to determine whether the effect was specific to Tau, or whether another aggregated protein, Aβ, elicited a similar response. We therefore assessed PTEN levels and activation in the Aβ-depositing APP23 mouse model [55]. Interestingly, western blot analysis of total protein isolated from whole brains of 12 month-old APP23 mice revealed a significant decrease in PTEN activation in APP23 compared to WT mice, with no change in total PTEN levels between the two groups (Fig. S3f).

### Inhibition of PTEN with bpV reduces protein phosphatase activity without altering levels or phosphorylation of Tau

**Fig. 6:**
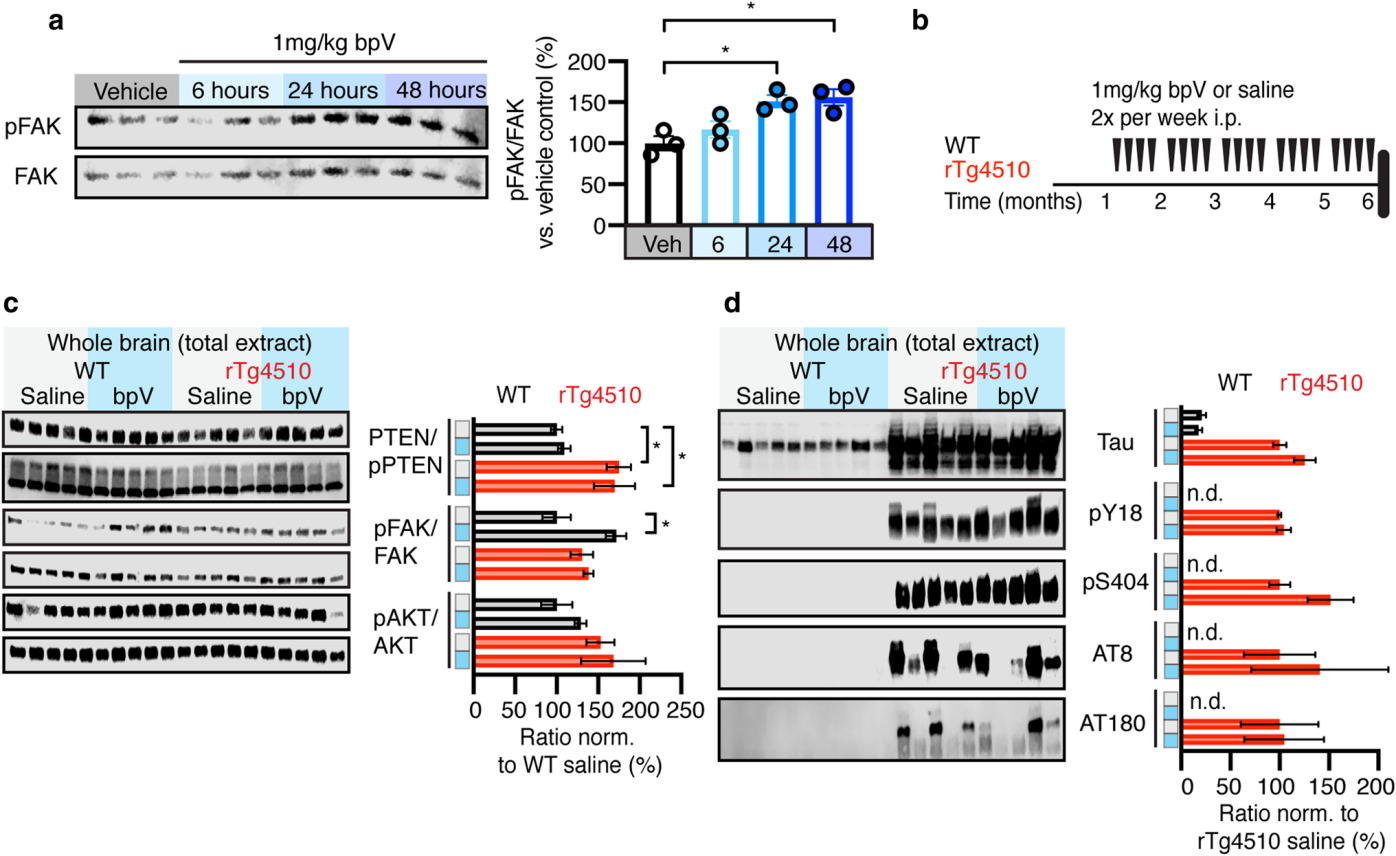
PTEN inhibition with bpV alters protein phosphatase pathway without reducing Tau levels or phosphorylation. **a** Representative western blots and quantification of pY397 FAK and total FAK in 6 month-old WT mice 6, 24, and 48 h after injection with bpV or saline control. **b** Study design of PTEN inhibition with bpV, injected intraperitoneally (i.p.) twice weekly for 5 months (starting at 1 month of age). **c** Representative western blots and quantification of pPTEN relative to PTEN, pY397 FAK relative to total FAK, and pS473 AKT relative to total AKT in brain lysates from saline- or bpV-treated rTg4510 and WT mice. The quantified ratio is expressed as a percent normalized to the saline-treated WT group. **c** Representative western blots and quantification of total Tau relative to total protein and pY18, pS404, pS202/T205 and pT231 each relative to total Tau in brain lysates from saline- or bpV-treated rTg4510 and WT mice. The quantified ratio is expressed as a percent normalized to the saline-treated rTg4510 group. Data presented as mean ± SEM, *p < 0.05, **a** one-way ANOVA with Tukey’s multiple comparison test, **b**, **c** two-way ANOVA with Tukey’s multiple comparison test

To confirm the involvement of PTEN in the neuron and synapse loss seen in the rTg4510 mice, we chose to inhibit PTEN pharmacologically using bisperoxovanadium(pic) (bpV). This compound is a protein tyrosine phosphatase (PTPase) inhibitor that readily crosses the blood–brain barrier [44] and that was shown to inhibit neuronal PTEN after systemic application, reducing apoptosis in development and under pathological conditions such as ischemia, trauma and oxidative stress [60]. As PTEN’s PTPase ability has been shown to be required for its ability to promote synapse loss [67], we sought to determine whether chronic inhibition of PTEN would be effective in preventing synapse loss in rTg4510 mice. We first validated the effect of bpV by intraperitoneally (i.p.) injecting 1 mg/kg bpV or saline as a control into 6 month-old WT mice and determined the phosphorylation of focal adhesion kinase (FAK), a known substrate of PTEN’s PTPase activity [59, 66], after 6, 24, and 48 h. We found that levels of pY397 FAK were increased at both the 24 and 48 h time-points relative to the saline control (Fig. 6a). We therefore delivered bpV twice weekly by i.p. injections (1 mg/kg), over a 5 month period (Fig. 6b).

At the end of the chronic treatment with bpV, mice were sacrificed 48 h after the final injection, followed by analysis of several components of the PTEN lipid and protein phosphatase pathways. As expected, we found that PTEN inhibition by bpV did not change its phosphorylation status (Fig. 6c). In the protein phosphatase pathway, we found that FAK phosphorylation at Y397 was only increased in the bpV-treated WT group; however, as this protein is a substrate of PTEN, we reasoned that the increased activation of PTEN counteracts the change seen after inhibition by bpV and that there are multiple tyrosine phosphorylation sites on FAK that could be further analyzed. In the PTEN lipid phosphatase pathway we saw no changes to pS473 AKT (Fig. 6c). To determine if PTEN inhibition would affect Tau levels and phosphorylation an immunoblot analysis of total brain extracts from bpV- and saline-treated rTg4510 and WT mice was performed. This revealed neither a change in total Tau relative to total protein nor phosphorylation of Tau as evaluated for a range of pathological epitopes (Y18, S404, S205/T205 and T231) relative to total Tau (Fig. 6d), suggesting that these pathological Tau phosphorylation sites are upstream of PTEN. Based on these findings we conclude that chronic treatment with bpV over a 5 month-period intermittently reduces PTEN’s PTPase activity in WT and rTg4510 mice and does not affect Tau levels or phosphorylation.

### Inhibition of PTEN reduces loss of neurons and synapses in rTg4510 mice

**Fig. 7:**
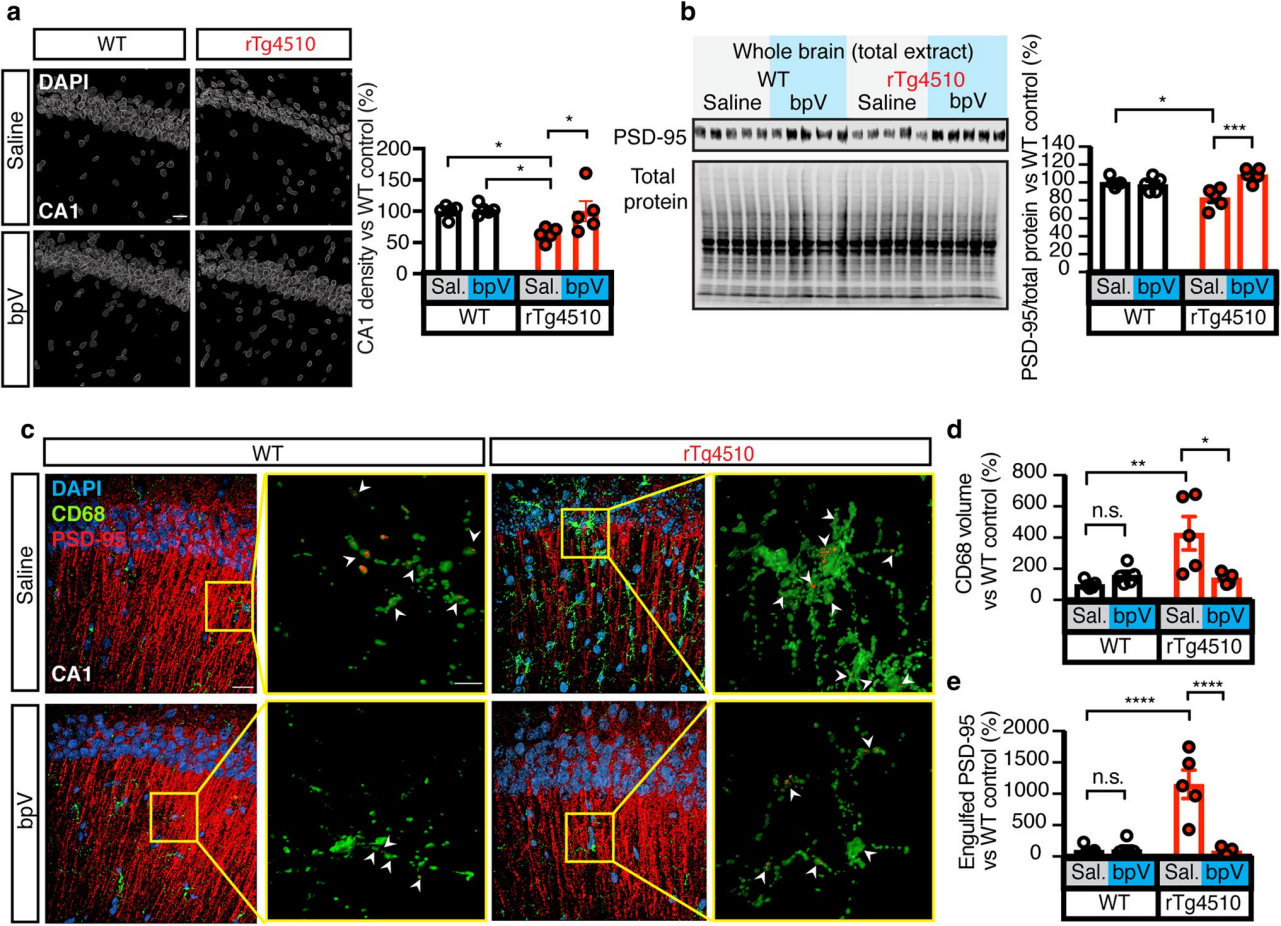
PTEN inhibition prevents microglia mediated cell and synapse loss in rTg4510 mice. **a** Surface rendering of DAPI from all groups, showing a rescue in cellular density of the cellular layer of the CA1 region of the hippocampus following chronic PTEN inhibition. **b** Representative western blots and quantification for PSD-95 and total protein in brain lysates from saline- or bpV-treated rTg4510 and WT mice.), revealing increased PSD-95 levels in bpV- compared to saline-treated rTg4510 mice. **c** 3D renderings of PSD-95 (red), CD68 (green) and DAPI (blue) in the hippocampus of saline- or bpV-treated rTg4510 and WT mice; arrowheads indicate engulfment. Scale bar in composite images: 20 μm; in zoomed-in images: 5 μm. **d** Quantification of CD68 volume, indicating that there is significantly less CD68 in rTg4510 mice treated with the PTEN inhibitor bpV. **e** Quantification of PSD-95 engulfed by microglia indicates that the rescue of PSD-95 loss in the bpV-treated group is due to a decrease in microglial engulfment. Data presented as mean ± SEM, *p < 0.05, **p < 0.01, ***p < 0.001, ****p < 0.0001; **a**, **b**, **d**, and **e** two-way ANOVA with Tukey’s multiple comparison test

The major functional outcome we sought to investigate was the effect PTEN inhibition has on synapse and neuron loss. We therefore quantified the number of pyramidal cell nuclei (using DAPI) in the CA1 region. This revealed that bpV treatment prevented neuronal loss in rTg4510 mice (Fig. 7a), whereas no changes were observed in WT mice. We next quantified levels of PSD-95 by immunoblotting, revealing a significant increase in bpV-treated rTg4510 mice (Fig. 7b). Again, WT mice exhibited no difference in response to bpV versus saline treatment (Fig. 7b). The bpV-treated rTg4510 mice also displayed a significantly reduced volume of CD68-positive lysosomes (Fig. 7c, d). We performed high resolution spinning-disk confocal microscopy followed by surface rendering to quantify the amount of PSD-95 internalized by CD68-positive lysosomes, revealing significantly less PSD-95 in lysosomes from bpV-treated compared to saline-treated rTg4510 mice (Fig. 7c, e). These data provide evidence that PTEN inhibition prevents microglia-mediated synapse loss in rTg4510 mice, and that PTEN acts downstream of Tau.

### PTEN activation displays dichotomous relationship with age in humans and mice

**Fig. 8:**
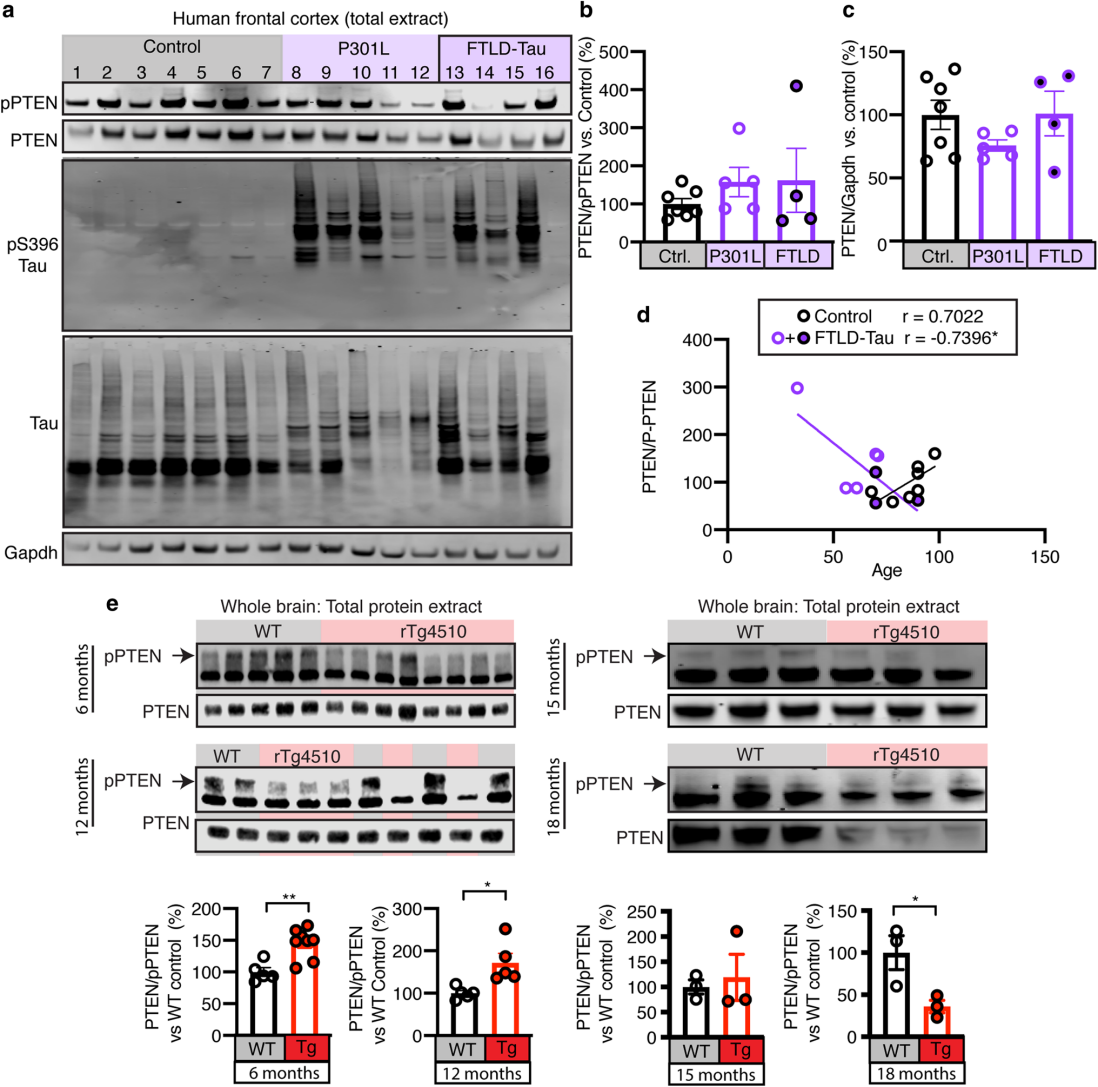
PTEN activation negatively correlates with age in human FTLD-Tau patients. **a** Representative western blots of total protein extracts from the frontal cortex of human P301L mutation carriers, sporadic FTLD-Tau patients and healthy controls for pPTEN, PTEN, pS396 Tau, total Tau and Gapdh. **b** Quantification of PTEN activation (PTEN/pPTEN) from **a**. **c** Quantification of PTEN levels relative to Gapdh from **a**. **d** Correlation between PTEN/pPTEN and age in healthy control and combined familial and sporadic FTLD-Tau patients, showing a negative relationship between age and PTEN activation in disease patients (*r* = − 0.7396, *p = 0.0360) and a positive correlation in healthy controls (*r* = 0.7022, *p* = 0.0786). One outlier was identified in the FTLD-Tau group and removed from the analysis. **e** Representative western blots and quantification of inactive pPTEN and total PTEN in total protein lysates from whole brains of 6, 12, 15, and 18 month-old WT and rTg4510 mice, showing that PTEN activation begins at 6 months of age, peaks between 12 and 15 months and is decreased at 18 months relative to WT controls. Data presented as mean ± SEM, *p < 0.05, **p < 0.01; **b**, **c** one-way ANOVA with Tukey’s multiple comparison test, **d** Pearson correlation, **e** unpaired *t* tests

PTEN accumulates in AD brains bearing neurofibrillary tangles [50]; however, what the activation status is of PTEN in FTLD-Tau brains is not known. Therefore, we assessed the ratio of total PTEN to pPTEN as a measure of activity in FTLD-Tau brain samples, P301L mutation carriers and healthy controls, revealing a non-statistically significant trend in increased active PTEN in the frontal cortex of diseased brains compared to healthy samples (Fig. 8a, b), whereas total levels did not differ between the three groups (Fig. 8a, c). To account for the high level of variability in our samples, we plotted the PTEN/pPTEN ratio against age for combined familial and sporadic disease patients and healthy controls. This revealed a negative relationship between age and PTEN activation in the disease group, and a positive relationship in the control group (Fig. 8a, d).

Having observed a negative relationship between age and PTEN activation in FTLD-Tau patient brains we hypothesized that there may be early and late stage differences in PTEN activation during tauopathy. To determine if PTEN activation rises early in disease pathogenesis and falls at later stages, we analyzed additional age groups of the rTg4510 line. In total protein extracts from whole brains of 6 and 12 month-old rTg4510 and WT mice, PTEN activation was significantly increased in the rTg4510 mice at both time points (Fig. 8e). At 15 months, we found comparable PTEN activation in the two genotypes, whereas at 18 months of age, activation decreased significantly in the rTg4510 mice (Fig. 8e), with the later time points more closely resembling what we had found in FTLD-Tau patient brains.

## Discussion

Phosphatase and tensin homolog (PTEN) regulates synaptic density during brain development [29]. Here, we reveal that PTEN has also a critical role in the neuronal and synaptic loss that characterizes tauopathy [15]. Using the tauopathy mouse model rTg4510, we found a significant decrease in PTEN phosphorylation, indicative of its activation [67] in neurons and synapses. This activation of PTEN preceded the apoptotic signatures caspase-3 cleavage and PS exposure, and was followed by microglial phagocytosis of neuronal structures. We further validated our PTEN findings in an additional tauopathy strain, the K3 mice, and confirmed that the effect was specific to Tau, as increased PTEN activation was observed in K3 mice, but not in Aβ plaque-forming APP23 mice. Moreover, chronic administration of the PTEN inhibitor bpV over 5 months to rTg4510 mice alleviated the synaptic and neuronal degeneration, as well as decreased microglial phagocytosis of synapses. Although several studies have shown that PTEN regulates synaptic density [29, 40, 67], our study is the first to reveal an involvement of PTEN in the process of synaptic elimination by microglia.

A recent study reporting that synapse loss in AD is mediated by complement and microglia has opened the door to obtain further mechanistic insight into this process [18]. Tau accumulates in the synapse, where it mediates the synaptotoxicity of Aβ [19, 22], and at the same time pathological Tau can, independent of Aβ, promote microglia- and complement-mediated synapse loss [5, 10, 32, 65]. Our data are in agreement with these studies by revealing that synapse loss is, at least in part, mediated by microglia. We propose a process by which PTEN has a role in microglial uptake of neuronal structures in the presence of pathological Tau phosphorylation. In support, we preserved levels of PSD-95 in rTg4510 mice after chronic treatment with the PTEN inhibitor bpV initiated prior to the occurrence of synapse loss. As the complement pathway functions in development and tauopathies to regulate synapse numbers [10, 18, 53, 54], PTEN may function similarly in tauopathy as it also regulates synapse numbers during development [29, 40, 64].

Recent evidence shows that physiological Tau restricts PTEN’s activity, and that PTEN activity could increase as a result of a loss of function of Tau due to its aggregation [33]. In support of this notion, Tau has been shown to interact with other phosphatases, such as protein phosphatase 2A, through its proline-rich domain, which is also recognized by Fyn [51]. During the revision of our manuscript, a study has been published which shows that Tau indeed interacts with PTEN via Tau’s proline-rich domain [57]. As disease-relevant phosphorylation or FTLD mutations of Tau can increase the association with Fyn [3, 31], these pathological changes may release PTEN from being restricted by Tau. We found that PTEN inhibition by bpV neither affected levels nor phosphorylation of Tau, indicating that PTEN functions downstream of Tau in mediating cellular and synaptic loss.

Regarding PTEN’s lipid phosphatase pathway, which has primarily been studied in Aβ rather than Tau models, we found that phosphorylation of the pro-survival pathway component AKT, did not differ between groups. Previously, the PTEN lipid phosphatase pathway has been shown to be dysregulated in APP/PSEN1 mice, leading to cognitive impairment [28]. In APP/PSEN1 mice, PTEN-regulated cognitive impairment presents as increased synaptic long-term depression, which is ameliorated by inhibition of PTEN with VO-OHpic, or the synthetic peptide PTEN-PDZ, which disrupts the binding of PTEN to PSD-95 [28]. It is well established that PTEN’s lipid phosphatase activity is required for its function in synaptic long-term depression, which may mediate synaptic dysfunction [1, 24, 28], however PTEN’s ability to induce spine loss is dependent on its protein phosphatase ability [67]. This may indicate that PTEN functions through its protein phosphatase activity to induce synapse loss which is considered to be upstream of cell loss in tauopathy, and protein substrates of PTEN could serve as potential therapeutic targets.

Targets of PTEN’s protein phosphatase activity that may function in the process of cell and synapse loss are FAK and insulin receptor substrate 1 (IRS1) [48, 59]. It has been shown in Tau knockout mice, that PTEN is activated, leading to neuronal insulin resistance through PTEN dephosphorylation of IRS1 [33]. Neuronal insulin resistance, which can occur in tauopathy, has been shown to lead to neuronal dysfunction and synapse loss [6]. We found that inhibition of PTEN by bpV resulted in increased phosphorylation of FAK, which functions in the formation of dendritic spines through Rho family GTPases, a class of signaling G proteins shown to be downregulated in tauopathy [10, 39].

Our results focus on neuronal mechanisms elicited by PTEN inhibition; however, it is conceivable that PTEN functions in other cell types of the brain, such as microglia, also contribute to disease progression. Neuronal loss in tauopathy mice is facilitated by microglia [47]. Under pathological conditions, microglia release pro-inflammatory compounds such as nitric oxide and tumor necrosis factor-α that have toxic effects on neurons and oligodendrocytes, effects that can be reduced by PTEN inhibition [62]. It has also been shown that deletion of neuronal PTEN is sufficient to induce de novo myelination [14], and that myelin degeneration contributes to age-related cognitive decline [61].

In the current study, we have demonstrated that PTEN is associated with the degenerative process of tauopathy, and we revealed that inhibition of PTEN can, at least in part, rescue this process. We provided evidence that increased PTEN activation occurs in mouse models of tauopathy, promoting synapse loss. These changes to PTEN occur together with an increased presence of apoptotic markers and synaptic elimination by microglia. Finally, we provided evidence for a role of PTEN in synapse loss by chronic pharmacological inhibition of PTEN with the PTPase inhibitor bpV which reduced microglial engulfment of synapses. Considering that PTEN is a tumor suppressor, we caution that PTEN may not be a viable therapeutic target itself, whereas neuron- or synapse-specific substrates of its protein phosphatase activity may turn out to be safer alternatives. Thus, the synthetic PTEN-PDZ peptide which disrupts the interaction of PTEN and PSD-95 may present a more suitable therapeutic option than inhibitors of PTEN’s enzymatic activity [27, 28]. In conclusion, our study adds PTEN to a growing number of proteins whose activity has seemingly opposing roles in diseases such as cancer [7, 17] and Tau-driven neurodegeneration.

## Electronic supplementary material

Below is the link to the electronic supplementary material.

Electronic supplementary material 1 (PDF 1197 kb)

Electronic supplementary material 2 (DOCX 21 kb)
