# Supplementary material for: PTEN activation contributes to neuronal and synaptic engulfment by microglia in tauopathy
Source: Acta Neuropathol. 2020 Mar 31;140(1):7–24. doi: 10.1007/s00401-020-02151-9 (PMC7300099; doi:10.1007/s00401-020-02151-9)

Supplemental Table 1 Reagent and resource list.

Reagent or Resource	Source	Identifier	
**Antibodies**			**Dilution**
PSD95 (Clone K28/43)	Millipore	RRID:AB_11212169	1:1000 WB
PSD95 (D27E11) XP	Cell Signalling	RRID:AB_2292883	1:500 IHC
PTEN (138G6)	Cell Signalling	RRID:AB_390810	1:1000 WB<br>1:100 IHC
Phospho-PTEN (Ser380/Thr382/383)	Cell Signalling	RRID:AB_331411	1:1000 WB
Cleaved Caspase-3 (Asp175) (5A1E)	Cell Signalling	RRID:AB_2070042	1:1000 IHC
C1q	Abcam	RRID:AB_2732849	1:1000 IHC
MAP2	Abcam	RRID:AB_2138147	1:1000 IHC
Total Tau	DAKO	RRID:AB_10013724	1:1000 WB
Total Human Tau (HT7)	ThermoFisher	RRID:AB_2314654	1:5000 WB
Phospho-Tyr18 Tau (Tyr18)	MediMabs	1944110227-P	1:1000<br>WB/IHC
Phospho-Ser396 Tau	Thermofisher	RRID:AB_2533745	1:1000 WB
Phospho-Tyr397 FAK	Cell Signalling	RRID:AB_10891442	1:500 WB
FAK	Cell Signalling	RRID:AB_2269034	1:500 WB
Phospho-Ser473 AKT	Cell Signalling	RRID:AB_2315049	1:1000 WB
AKT	Cell Signalling	RRID:AB_329827	1:1000 WB
CD68	BioRad	RRID:AB_324217	1:1000 IHC
AT8 Tau	ThermoFisher	RRID:AB_223647	1:1000 WB
AT180 Tau	ThermoFisher	RRID:AB_223649	1:1000 WB
Phospho-Ser404 Tau	ThermoFisher	RRID:AB_2532674	1:1000 WB
Iba1	Wako	RRID:AB_839504	1:1000 IHC
Cd11b	Abcam	RRID:AB_306831	1:1000 IHC
Anti-rat IgG (H+L), (Alexa Fluor® 488 Conjugate)	Cell Signalling	RRID:AB_10693769	1:1000 IHC
Goat anti-Mouse Alexa Fluor 488	ThermoFisher	RRID:AB_2534069	1:1000 IHC
Goat anti-Rabbit Alexa Fluor 555	ThermoFisher	RRID:AB_2536100	1:1000 IHC
Goat anti-Chicken Alexa Fluor 647	ThermoFisher	RRID:AB_2535866	1:1000 IHC
IR DYE 680RD Goat anti-Mouse	Licor	RRID:AB_10956588	1:10000 WB
IR DYE 800RD Goat anti-Rabbit	Licor	RRID:AB_621843	1:10000 WB
		-	
**Chemicals**			
pSIVA	Novous Bio	NBP2-29382	
Calcein AM Blue	ThermoFisher	C1429	
F2N12S	ThermoFisher	A35137	
pHrodo Red AM	ThermoFisher	P35372	
Dipotassium Bisperoxovanadium(pic) dihydrate	Sigma/Merck	SML0885	
Revert Total Protein Stain	Licor	926-11011	
VECTASHIELD Hardset antifade mounting medium,	Vector Labs	H-1400	
DMEM F12	ThermoFisher	11320033	

Live Cell Imaging Solution	ThermoFisher	A14291DJ	
Odyssey Blocking buffer in TBS	Licor	927-50000	
Neurobasal Medium	ThermoFisher	21103049	
B27 Supplement	ThermoFisher	17504044	
Lipofectamine 2000	ThermoFisher	11668019	
Lamda Phosphatase	NEB	P0753S	
**Experimental Models: Organisms/Strains**			
129S6.Cg-Tg(Camk2a-tTA)1Mmay/JlwsJ; Fgf14Tg(tetO-MAPT*P301L)4510Kha/J.	Jackson Laboratory	RRID:IMSR_JAX:024854	
B6.129P2(Cg)-*Cx3cr1tm1Litt*/J	Jackson Laboratory	RRID:IMSR_JAX:005582	
K3	In house	Ittner et al. PNAS 2008	
APP23	Jackson Laboratory	RRID:IMSR_JAX:030504	
**Software**			
Prism 7	GraphPad	RRID:SCR_002798	
FIJI	NIH	RRID:SCR_002285	
Imaris 9.4.2	Bitplane	RRID:SCR_007370	
Image Studio Software	LI-COR	RRID:SCR_015795	
Huygens Deconvolution	Scientific Volume Imaging	RRID:SCR_014237	

Supplemental Figure 1

a

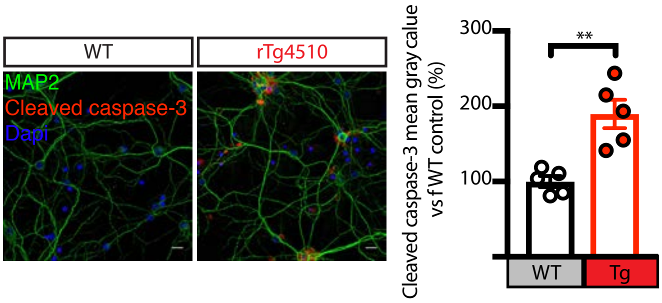

Supplemental Figure 2

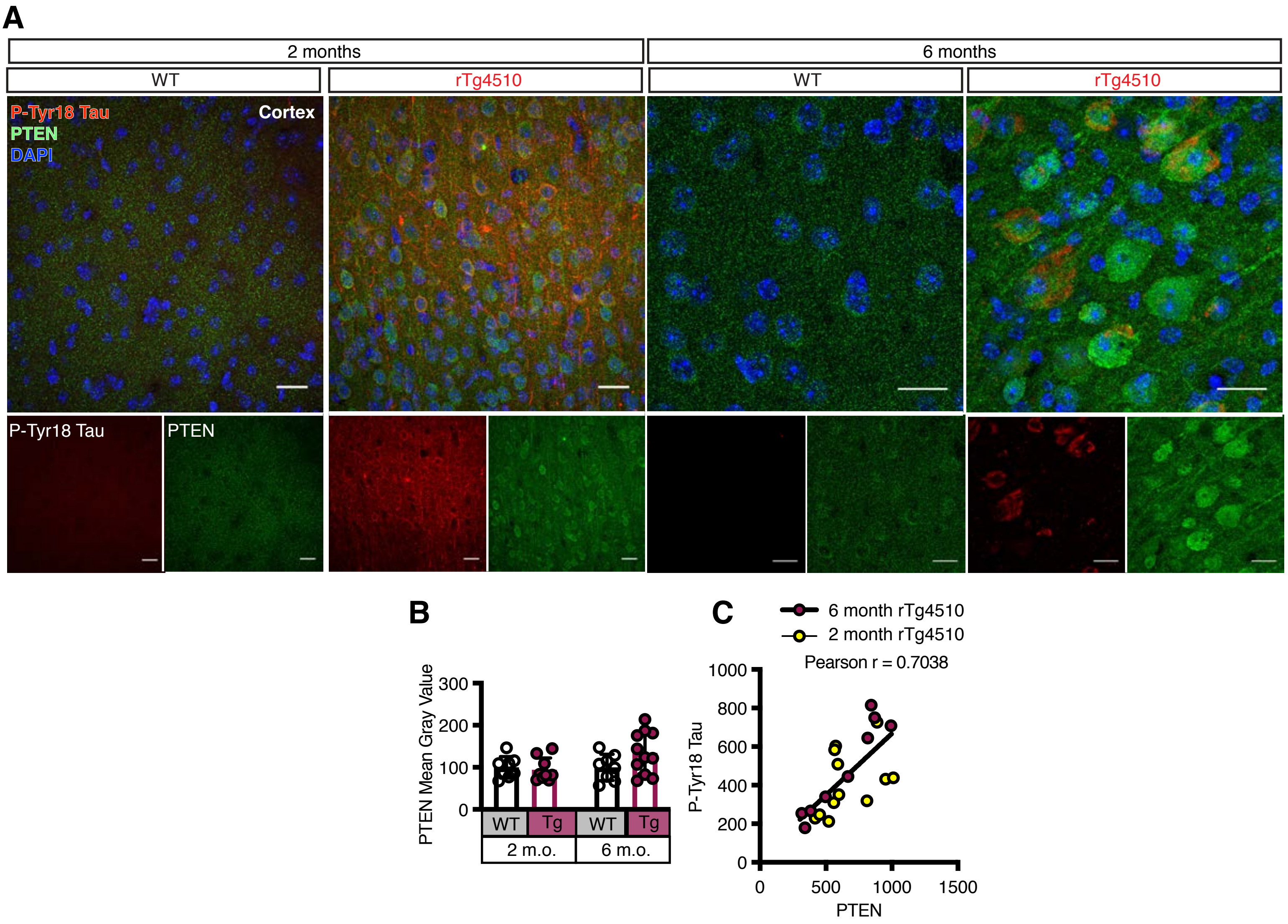

Supplemental Figure 3

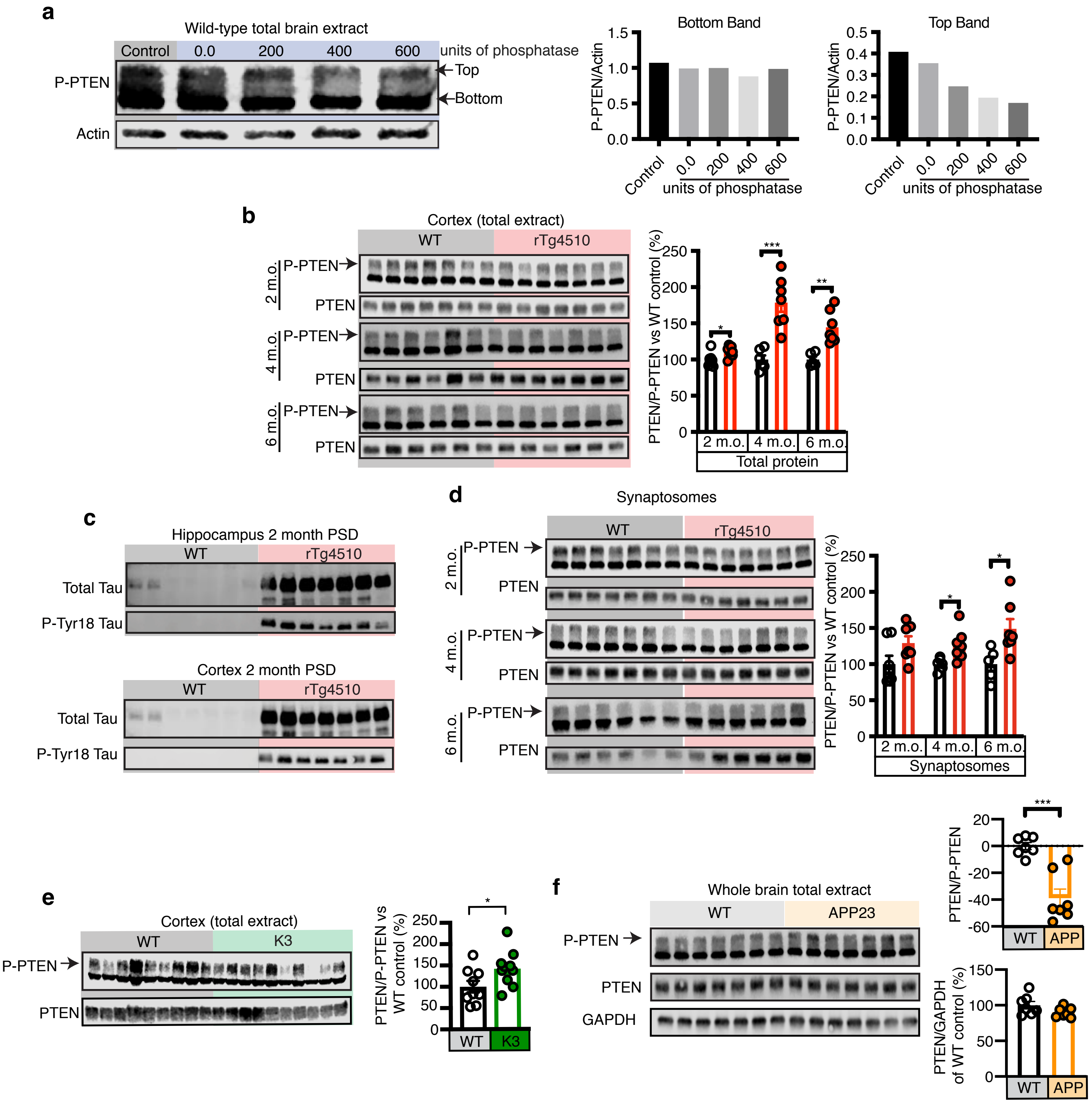

Supplemental figure 4

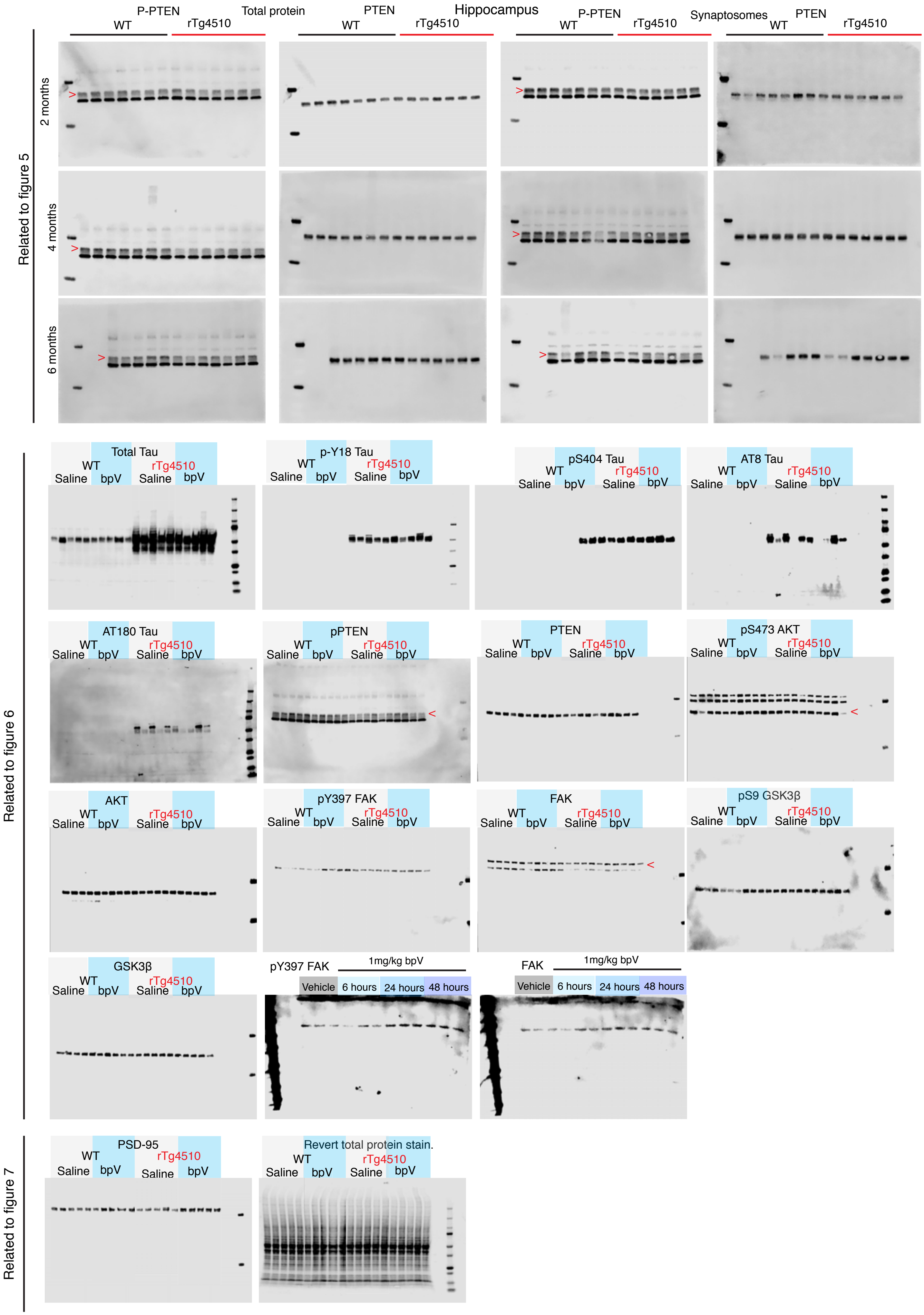

Supplemental figure 5

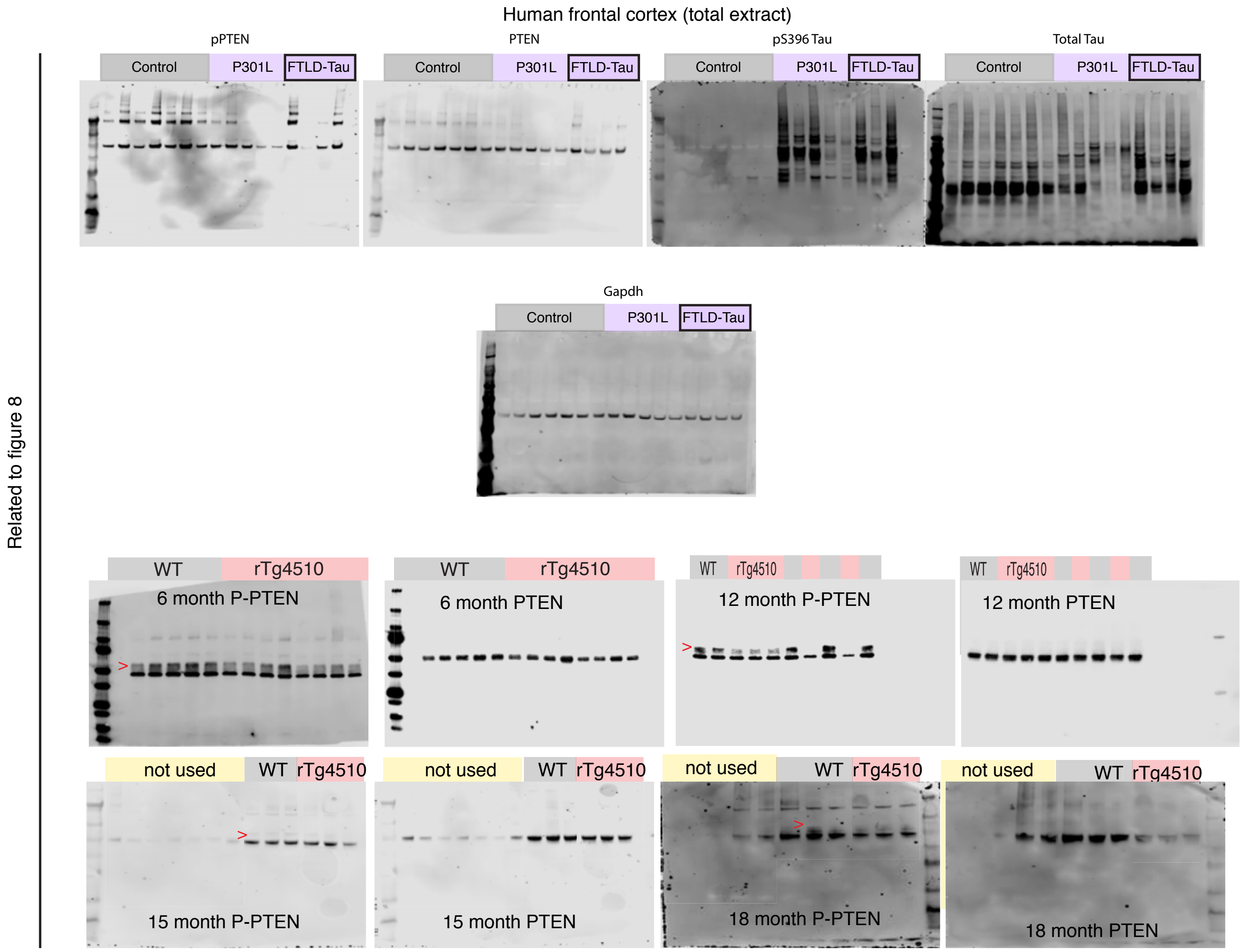

Supplement: Supplementary file 1 — Electronic supplementary material 1 (PDF 1197 kb) [file 401_2020_2151_MOESM1_ESM.pdf]
