# Supplementary material for: PTEN activation contributes to neuronal and synaptic engulfment by microglia in tauopathy
Source: Acta Neuropathol. 2020 Mar 31;140(1):7–24. doi: 10.1007/s00401-020-02151-9 (PMC7300099; doi:10.1007/s00401-020-02151-9)
Supplement: Supplementary file 2 — Electronic supplementary material 2 (DOCX 21 kb) [file 401_2020_2151_MOESM2_ESM.docx]

**Supplemental Fig. 1** Confirmation of apoptosis in rT4510 primary neurons related to Fig. 1. **a** Images and quantification of cleaved caspase 3 (red), MAP2 (green) and DAPI (blue) from DIV 20 WT and rTg4510 primary neurons. Scale bar, 10 μm. Data presented as mean ± SEM, **p < 0.01; **a** unpaired t-test.

**Supplemental Fig. 2** PTEN and Tau correlation extends to cortical regions, related to Fig. 2.

**a** Representative fluorescence images for P-Tyr18 Tau (red), PTEN (green), and DAPI (blue) of 10 μm thick maximum intensity z-projections of the cortex of 2 and 6 month-old rTg4510 and WT mice. Scale bars: 20 μm. **b** Quantification of PTEN mean gray value in the cortex of 2 and 6 month old rTg4510 and WT mice. **c** Spearman correlation between the PTEN and P-Tyr18 tau mean gray values. Data presented as mean ± SEM; **b** two-way ANOVA with Tukey’s multiple comparison test, **c** Pearson correlation.

**Supplemental Fig. 3** Characterization of pPTEN and analysis of PTEN activation in multiple models of Tau and Aβ pathology, related to Fig 5. **a** Representative western blots and quantification showing the characterization of the corresponding P-PTEN band after treatment with phosphatase. Top and bottom P-PTEN bands individually quantified relative to Actin. **b** Representative western blots and quantification of inactive P-PTEN and total PTEN in total protein lysates from the cortex of 2, 4, and 6 month-old WT and rTg4510 mice, showing a smaller fraction of PTEN in the inactive form at 2, 4 and 6 months in total protein lysates **c** Western blots for HT7 (total human Tau) and P-Tyr18 Tau of hippocampal PSD-enriched lysates from hippocampus and cortex of 2 month-old rTg4510 and WT mice. **d** Representative western blots and quantification of inactive P-PTEN and total PTEN in synaptosomal lysates from the cortex of 2, 4, and 6 month-old WT and rTg4510 mice, showing a smaller fraction of PTEN in the inactive form at 4 and 6 months in synaptosomes. **e** Representative western blots and quantification of P-PTEN and total PTEN using extracts from the cortex of 12 month-old K369I tau transgenic K3 mice showing an increase in PTEN activation. **f** Representative western blots of total protein extracts from the whole brain of 12 month old APP23 and WT mice for P-PTEN, PTEN and Gapdh .Quantification of the ratio of total PTEN relative to inactive phosphorylated PTEN P-PTEN. Quantification of the ratio of total PTEN relative to total Gapdh Data presented as mean ± SEM, *p < 0.05, **p < 0.01,***p <0.001; **b, d, e, and f** unpaired t-tests.

**Supplemental Fig. 4** Western blot images, related to Fig. 5, 6, and 7.

Full western blots from all primary data experiments.

**Supplemental Fig. 5** Western blot images related to Fig. 8.

Full western blots from all supplemental data experiments.
